# Acute Generalized Exanthematous Pustulosis Associated With Herpes Simplex Virus Reactivation Due to Cold Medicine: A Case Report and Review of the Literature

**DOI:** 10.7759/cureus.80117

**Published:** 2025-03-05

**Authors:** Yoshihito Mima, Tsutomu Ohtsuka, Nobuhito Arakawa, Yoshimasa Nakazato, Yuta Norimatsu

**Affiliations:** 1 Department of Dermatology, Tokyo Metropolitan Police Hospital, Tokyo, JPN; 2 Department of Dermatology, International University of Health and Welfare Hospital, Tochigi, JPN; 3 Department of Respiratory Medicine, International University of Health and Welfare Hospital, Tochigi, JPN; 4 Department of Diagnostic Pathology, International University of Health and Welfare Hospital, Tochigi, JPN; 5 Department of Dermatology, International University of Health and Welfare, Narita Hospital, Narita, JPN

**Keywords:** acute generalized exanthematous pustulosis, cd4-positive t cell, cold medicine, drug-induced eruptions, herpes simplex virus

## Abstract

Acute generalized exanthematous pustulosis (AGEP) is a severe skin inflammation characterized by the sudden onset of numerous sterile, non-follicular pustules on an erythematous and edematous background, usually associated with fever. AGEP is commonly triggered by medications such as antibiotics. However, an association between AGEP and viral infections has also been reported recently. We report a case of a 70-year-old man who developed AGEP following the intake of an over-the-counter cold medicine in the context of herpes simplex virus (HSV) reactivation. Furthermore, we review three cases of AGEP associated with HSV infection. This report suggests HSV reactivation as a potential trigger for AGEP, emphasizing the need for caution when administering drugs to patients with HSV infection.

## Introduction

Acute generalized exanthematous pustulosis (AGEP) is a condition characterized by the acute onset of diffuse edematous erythema, particularly in intertriginous areas and the face, with numerous non-follicular, sterile pustules developing on erythematous skin. Patients may experience associated burning sensations or pruritus. The majority of AGEP cases present with fever exceeding 38°C and leukocytosis, often accompanied by mild eosinophilia [1]. AGEP is commonly triggered by medications, including antibiotics, anticancer agents, and hydroxychloroquine, typically occurring within a week of exposure to the causative drug [2]. Clinically, the pustules of AGEP resolve spontaneously within 4-10 days, often leaving behind desquamation and post-inflammatory hyperpigmentation. The prognosis is generally favorable, but in elderly or immunocompromised patients, high fever or secondary bacterial infections can pose life-threatening risks [1]. Several conditions require differential diagnosis from AGEP, including drug reaction with eosinophilia and systemic symptoms (drug-induced hypersensitivity syndrome), toxic epidermal necrolysis, subcorneal pustular dermatosis, generalized pustular psoriasis, bacterial folliculitis, Kaposi varicelliform eruption, Sweet’s syndrome, and Behçet’s disease [1]. Histopathologically, AGEP is characterized by spongiform subcorneal or intraepidermal pustules, prominent dermal papillary edema, and perivascular neutrophilic infiltration, with occasional eosinophil degranulation [3,4]. In some cases, perivascular neutrophilic infiltration and keratinocyte necrosis suggestive of vasculitis may be observed. However, epidermal hyperplasia, a hallmark of psoriasis, is typically absent [3,4]. The diagnosis of AGEP is based on clinical presentation and histopathological findings, with the European Severe Cutaneous Adverse Reactions (EuroSCAR) scoring system serving as a useful diagnostic tool [5]. The EuroSCAR score, proposed by Sidoroff A et al., is primarily used to distinguish AGEP from other differential diagnoses and to confirm the diagnosis. It includes categories such as morphology (pustules, erythema, distribution, desquamation post-pustulation), course (mucosal involvement, acute onset, resolution, fever, white blood cell count), and histopathology (skin biopsy). A EuroSCAR score of 8 or higher is considered positive and confirms the diagnosis of AGEP [5]. Additional tests such as patch testing or drug-induced lymphocyte stimulation tests (DLST) may be considered as needed [2]. The pathogenesis of AGEP involves multiple immune pathways. Activated CD4 and CD8 positive T cells respond to specific drug-related triggers, inducing keratinocyte apoptosis and tissue destruction, which leads to vesicle formation. Additionally, interleukin (IL)-8 or IL-36 secretion promotes neutrophil recruitment, contributing to pustule formation and peripheral neutrophilia [6]. The first-line treatment for AGEP is topical corticosteroids therapy and immediate discontinuation of the causative drug. In severe cases, oral prednisolone is recommended, while supportive therapy with antipyretics, and antihistamines can be beneficial. If systemic corticosteroid therapy is ineffective, cyclosporine may be considered as an alternative treatment [1,2].

Recently, associations between viral infections, including Epstein-Barr virus, parvovirus B19, and cytomegalovirus, and the development of AGEP have been reported. Furthermore, viral vaccinations, such as for COVID-19, can also contribute to the occurrence of AGEP [2,7,8].

Herein, we report a case of AGEP associated with herpes simplex virus (HSV) reactivation triggered by cold medicine intake, and review four cases of AGEP that occurred during HSV infection [9-10].

## Case presentation

**Figure 1:**
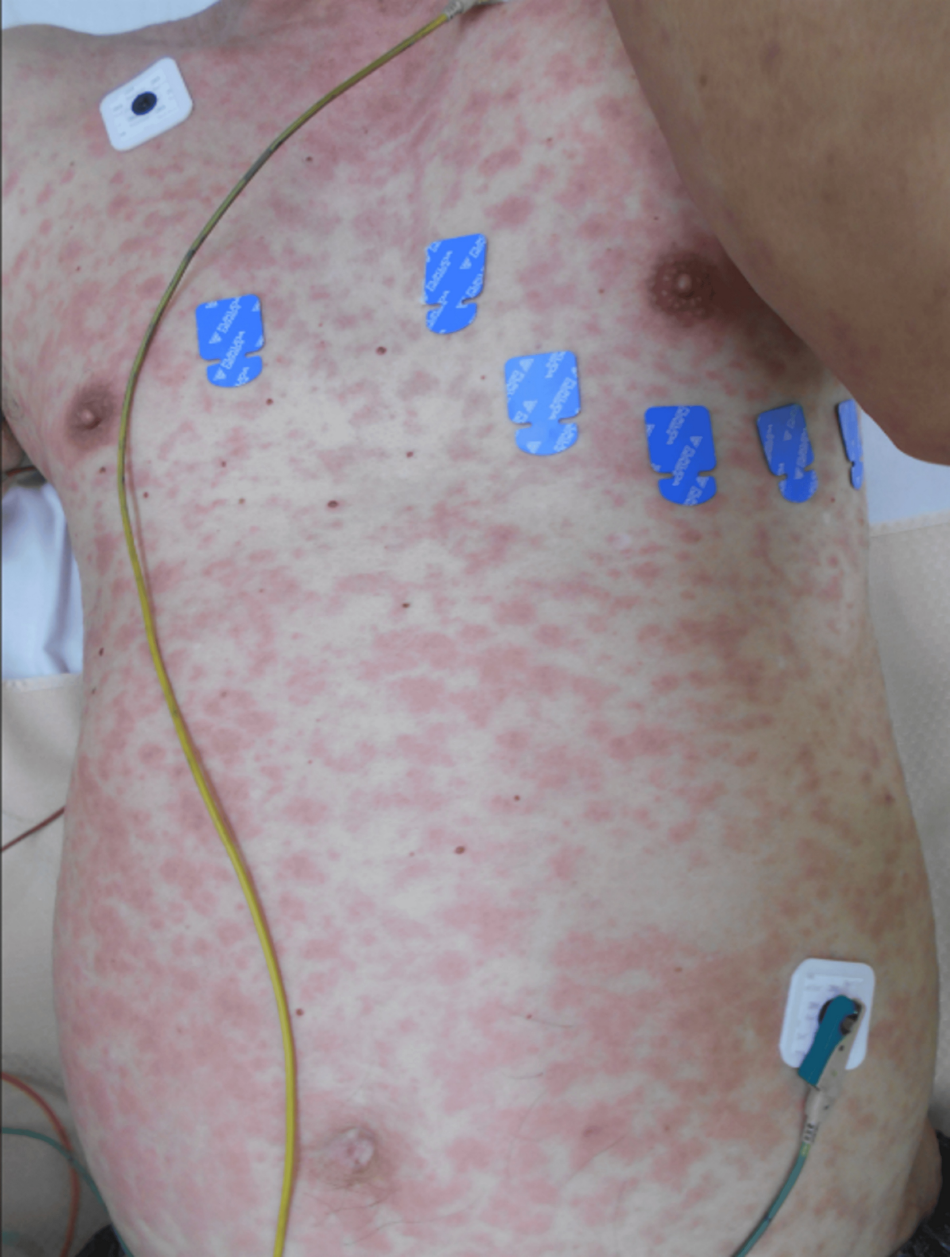
Physical examination revealing edematous, erythematous plaques and a disseminated pattern of erythema multiforme-like lesions on the trunk and extremities.

A 70-year-old man with no significant medical history or regular medication use developed a skin rash over his entire body six hours after taking an over-the-counter cold medicine containing acetaminophen, dihydrocodeine phosphate, ephedrine hydrochloride, and licorice. He had used it frequently in the past without any adverse effects. Despite discontinuing the medication, the rash progressively worsened over two days, prompting his visit to the emergency department. Physical examination revealed edematous, erythematous plaques and erythema multiforme-like lesions with a disseminated pattern on the trunk and extremities. No pustules, vesicles, or mucosal involvement were noted (Figure 1).

The patient exhibited hypotension (77/59 mmHg), hypoxia (84% on room air), and fever (38.6 °C). Laboratory examination revealed an elevated neutrophil count (13,600/μL, normal range: 3,500-9,000 /μL), a normal eosinophil count (272/μL), an increased C-reactive protein level (16.9 mg/dL, normal range: 0-0.3 mg/dL), and an elevated creatinine level (1.25 mg/dL, normal range: 0.6-1.0 mg/dL), indicating acute renal dysfunction. Blood smear analysis showed 9% abnormal lymphocytes. Urinalysis and computed tomography scans were unremarkable. Due to the severity of the eruption, the patient was urgently hospitalized and referred to the dermatology department the next day. Initially, a viral-related rash was suspected, and serological tests for viral antibodies were performed. Blood tests were positive for HSV immunoglobulin (Ig)G (49.9 enzyme immunoassay (EIA), normal range: 0-1.0 EIA) and IgM (2.21 EIA, normal range: 0-1 EIA), but negative for cytomegalovirus and Epstein-Barr virus. The elevation of CRP and WBC is consistent with inflammation caused by AGEP, while the increase in atypical lymphocytes suggests HSV reactivation. Furthermore, it was considered that AGEP-induced severe inflammation, leading to shock vital signs and circulatory impairment, contributed to renal dysfunction. All laboratory data is shown in Table 1.

**Table 1:** Results of laboratory examination. RR: Reference range; AST: Aspartate aminotransferase; ALT: Alanine aminotransferase; LDH: Lactate dehydrogenase; CRP: C-reactive protein; BUN: Blood urea nitrogen; Hb: Hemoglobin; IgG: Immunoglobulin G; IgM: Immunoglobulin M; CMV: Cytomegalovirus; EB VCA: Epstein-Barr virus viral capsid antigen; HSV: Herpes simplex virus.

Variables	Patient value	RR, adults
AST	33 U/L	11-33 U/L
ALT	73 U/L	6-37 U/L
Albumin	3.3 g/dL	3.8-5.0 g/dL
Total bilirubin	1.1 mg/dL	0.2-1.2 mg/dL
LDH	344 U/L	135-214 U/L
CRP	16.9 mg/dL	<0.50 mg/dL
BUN	22.1 mg/dL	8-20 mg/dL
Creatinine	1.25 mg/dL	0.6-1.0 mg/dL
WBC	13,600 /μL	3,500-9,000 /μL
Hb	13.9 g/dL	12-16 g/dL
Lactate	2.4 mmol/L	0-2 mmol/L
CMV IgM	Non-reactive	Non-reactive
CMV IgG	Non-reactive	Non-reactive
EB VCA-IgM	Non-reactive	Non-reactive
EB VCA-IgG	Reactive	Non-reactive
HSV IgM	Reactive	Non-reactive
HSV IgG	Reactive	Non-reactive
Abnormal lymphocyte	9.0%	0-5%

**Figure 2:**
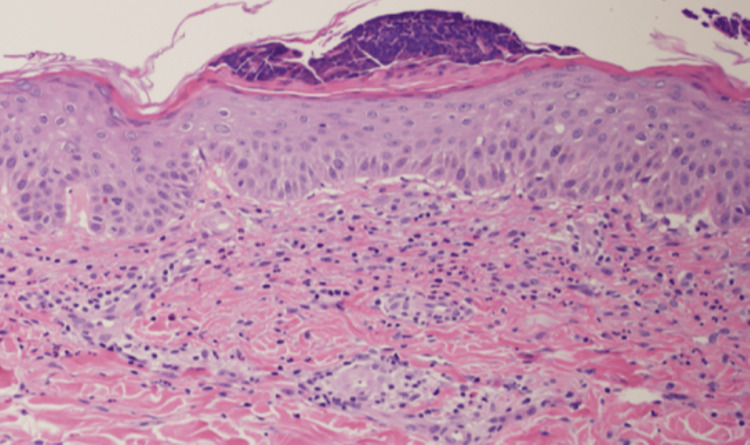
Histopathological examination revealing pustules with neutrophil aggregation in the subcorneal region, significant vacuolar degeneration, and marked infiltration of lymphocytes and eosinophils.

Histopathological examination revealed subcorneal pustules, liquefactive degeneration, lymphocytic infiltration into the basal layer, and perivascular inflammatory cell infiltration in the upper dermis (Figure 2).

**Figure 3:**
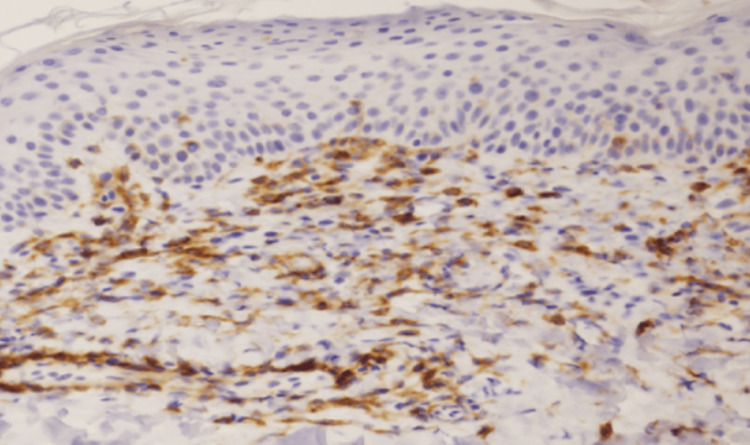
Immunostaining for CD4 reveals numerous CD4-positive T cells.

The lymphocytes infiltrating the upper dermis were predominantly composed of CD4-positive T cells (Figure 3); conversely, few were CD8-positive T cells infiltrating.

A DLST for the cold medicine yielded a strongly positive result (285%). The six-hour interval between drug ingestion and rash onset, absence of Nikolsky’s sign and mucosal symptoms, rapid resolution within two weeks, lack of psoriasis history, and a EuroSCAR score of 8 points (Table 2) all contributed to the diagnosis of AGEP induced by cold medicine with HSV reactivation.

**Table 2:** EuroSCAR score for the present case. EuroSCAR: European Severe Cutaneous Adverse Reactions.

Criteria	Description	Score
Morphology		
Pustules	Atypical	0
Erythema	Typical	2
Distribution	Typical	2
Postpustular desquamation	No	0
Course		
Mucosal involvement	No	0
Acute onset (<24 hours)	Yes	0
Resolution of pustules and erythema (<15 days)	Yes	0
Fever (>38℃)	Yes	1
Blood neutrophil count (>7000 μ/L)	Yes	1
Histopathology		
Skin biopsy	Subcorneal pustule with mild edema	2
Total score		8

Steroid pulse therapy had already been initiated by the department of internal medicine, and subsequent treatment in our department involved oral prednisolone (1.0 mg/kg/day) and betamethasone dipropionate ointment. The rash completely improved within two weeks without pigmentation, and treatment was tapered off within a month. There was no recurrence or complications of AGEP. Subsequent additional tests, including individual DLST and patch tests for each component of the combination over-the-counter cold medicine, were recommended. However, due to work and other reasons, the patient did not wish to undergo further testing, and no additional allergy tests were conducted.

## Discussion

Viral infections are known to independently trigger AGEP without drug involvement [11,12]. Additionally, they may interact with drug administration, promoting the onset of AGEP [7,8]. Typically, AGEP improves within a few days after discontinuation of the causative drug. However, in this case, the patient’s condition continued to deteriorate for two days despite stopping the medication, suggesting that both HSV reactivation and the administration of cold medicine contributed to the onset and exacerbation of AGEP. AGEP is characterized by a sterile neutrophilic inflammatory response [1,2]. Activated CD4+ and CD8+ T cells are believed to promote IL-8 and IL-36 production via IL-17 and IL-22, leading to neutrophil migration and pustule formation, which play central roles in AGEP pathogenesis [6-8]. Viral infections are known to immunologically stimulate CD4+ and CD8+ T cells [13]. Therefore, when a causative drug is administered in the presence of a viral infection, inflammatory CD4+ and CD8+ T cell infiltration into the skin may increase, resulting in excessive production of inflammatory cytokines such as IL-8 and IL-36, thereby triggering AGEP [6-8,13].

In this case, immunohistochemical staining revealed significant infiltration of CD4-positive T cells in the dermis, with some infiltrating into the basal layer of the epidermis. This finding suggests that the interaction between viral infection and drug exposure led to CD4+ T cell activation, triggering AGEP through excessive IL-8 and IL-36 production [6-8,13]. Since AGEP in this case was induced not only by drug exposure but also by viral infection, the inflammatory response may have been exacerbated, leading to a systemic inflammatory reaction severe enough to cause disturbances in vital signs, including hypotension and hypoxia.

To date, only three cases of AGEP associated with HSV reactivation have been reported (Table 3) [9,10].

**Table 3:** Three cases of AGEP associated with HSV. AGEP: Acute Generalized Exanthematous Pustulosis; HSV: Herpes Simplex Virus.

Case	Age	Sex	Past medical history	Diagnosis of HSV infection when AGEP was developed	History of taking causative drug	Onset time	Additional allergy test	Treatment for AGEP
Kubin ME et al. [9]	44	Woman	Atopic dermatitis and herpes labialis (clinically diagnosed)	Clinically diagnosed	Yes	One day after intake	Patch testing (+)	Steroid ointment and oral cephalexin
Serra D et al. [10]	53	Male	Herpetic retinitis (clinically diagnosed)	Clinically diagnosed	Yes	Three weeks after intake	Patch testing (+)	Foscarnet and methylprednisolone
Our case	70	Male	Herpetic labialis (clinically diagnosed)	Viral antibody test	Yes	Six hours after intake	Drug-induced lymphocyte stimulation test (+)	Steroid pulse therapy, followed by oral prednisolone and betamethasone dipropionate ointment

The patients were 44, 53, and 70 years old, comprising two males and one female. All cases developed AGEP within three days of drug administration. In the two previously reported cases, acyclovir was identified as the causative drug, and in addition to clinical course and histopathological findings, patch testing confirmed the diagnosis of AGEP associated with acyclovir. In contrast, in this case, cold medicine was implicated, and DLST test, along with clinical and histopathological findings, confirmed the diagnosis of AGEP associated with cold medicine. Ideally, patch testing or DLST should have been performed for each component of the cold medicine, including acetaminophen, dihydrocodeine phosphate, ephedrine hydrochloride, and licorice to precisely detect the causative drug. Although we proposed these tests, the patient’s work commitments and long travel distance made frequent hospital visits difficult. As a result, we concluded follow-up on the condition that the patient would avoid these suspicious components in the future. The inability to perform individual drug allergy tests remains a limitation of this report. Regarding treatment, the two previously reported cases improved with topical or oral corticosteroids [9,10]. In this case, steroid pulse therapy was initiated by the internal medicine department, leading to a rapid improvement in the patient’s overall condition within a few days. However, given the rapid resolution, it is possible that oral corticosteroids alone might have been sufficient, warranting a careful evaluation of the necessity of pulse therapy. Additionally, the absence of visible pustules may have been due to the timing of the assessment, as the first evaluation in our dermatology department occurred after steroid pulse therapy had already been administered.

This case highlights that even medications that were previously well tolerated can trigger AGEP in the setting of HSV infection, emphasizing the importance of carefully monitoring drug intake during viral infections. Although AGEP can be triggered by viral infections alone or through interactions between viral infections and causative drugs [2,7,8,11,12], its precise mechanisms remain unclear. Therefore, further studies and case accumulations are needed to elucidate the underlying pathophysiological pathways of AGEP.

## Conclusions

AGEP, a severe drug eruption, is characterized by the sudden onset of numerous small pustules on an erythematous and edematous background. The primary treatment involves discontinuing the causative drug and applying topical corticosteroids, with resolution typically occurring within two weeks. AGEP can be triggered by a viral infection alone or by the interaction between a viral infection and a causative drug. Herein, we present a case of AGEP induced by an over-the-counter cold medicine during HSV reactivation and review three previously reported cases of AGEP associated with HSV infection. This case underscores that even medications previously well-tolerated can induce AGEP in the presence of HSV infection. When a causative drug is administered during a viral infection, the increased infiltration of inflammatory CD4⁺ and CD8⁺ T cells into the skin may lead to excessive production of inflammatory cytokines such as IL-8 and IL-36, ultimately triggering AGEP. However, the precise mechanisms underlying this interaction remain unclear, necessitating further research and case accumulation to elucidate the pathophysiological pathways of AGEP.
